# Switching off malignant mesothelioma: exploiting the hypoxic microenvironment

**DOI:** 10.18632/genesandcancer.124

**Published:** 2016-11

**Authors:** Noushin Nabavi, Kevin L. Bennewith, Andrew Churg, Yuzhuo Wang, Colin C. Collins, Luciano Mutti

**Affiliations:** ^1^ Laboratory for Advanced Genome Analysis, Vancouver Prostate Centre, BC, Canada; ^2^ Department of Urologic Sciences, University of British Columbia, BC, Canada; ^3^ Department of Experimental Therapeutics, BC Cancer Agency, BC, Canada; ^4^ Department of Integrative Oncology, BC Cancer Agency, BC, Canada; ^5^ Department of Pathology and Laboratory Medicine, University of British Columbia, BC, Canada; ^6^ Italian Group for Research and Therapy for Mesothelioma (GIMe) & School of Environment and Life Sciences, University of Salford, Manchester, United Kingdom

**Keywords:** mesothelioma, hypoxia, metabolism, cell cycle, proteolysis, DNA damage, angiogenesis, solid tumors

## Abstract

Malignant mesotheliomas are aggressive, asbestos-related cancers with poor patient prognosis, typically arising in the mesothelial surfaces of tissues in pleural and peritoneal cavity. The relative unspecific symptoms of mesotheliomas, misdiagnoses, and lack of precise targeted therapies call for a more critical assessment of this disease. In the present review, we categorize commonly identified genomic aberrations of mesotheliomas into their canonical pathways and discuss targeting these pathways in the context of tumor hypoxia, a hallmark of cancer known to render solid tumors more resistant to radiation and most chemo-therapy. We then explore the concept that the intrinsic hypoxic microenvironment of mesotheliomas can be Achilles' heel for targeted, multimodal therapeutic intervention.

## Etiology and clinical manifestations of pleural mesotheliomas

**Figure 1:**
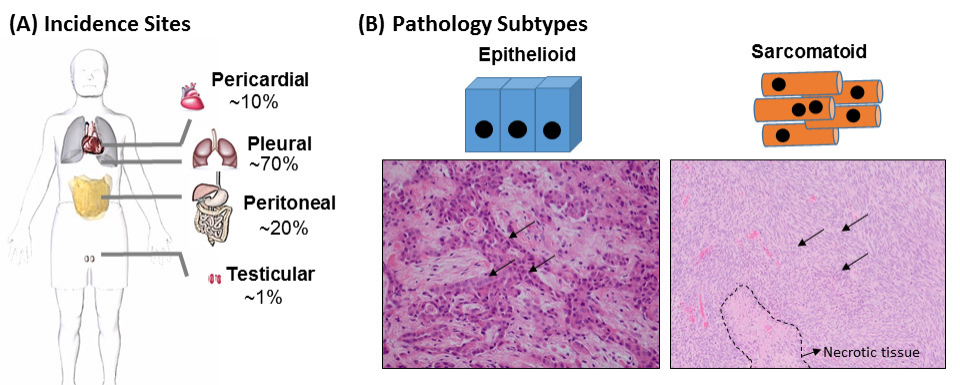
Common sites of incidence and pathological subtypes of pleural mesothelioma (A) Tissues affected by mesothelioma and incidence rates. (B) Hematoxylin and eosin staining of two mesothelioma pathologic subtypes (epithelioid and sarcomatoid). Biphasic phenotype is a mixture of epithelioid and sarcomatoid types. The arrows indicate disorganized neoplastic tumor areas.

Determining cancer etiology is an intricate process because evidence from genetics, cellular and molecular biology, as well as epidemiology and pathology must be integrated to gain a complete understanding of carcinogenicity [1]. Malignant mesotheliomas (derived from the Greek word mésos “middle” and oma “tumor”) are rare cancers that originate from deregulated cellular proliferation of the mesoderm tissue lining the chest cavity, heart, lungs, the abdominal cavity, and the intra-abdominal organs [2, 3]. Scans made via computed tomography (CT), magnetic resonance image (MRI), or positron emission tomography (PET) are required to determine the location and extent of the disease [4]. More than 70% of the diagnosed cases are pleural, 20% are peritoneal, and less than 1% are pericardial or testicular types (as depicted in Figure 1A) [5-7].

The annual incidence of malignant mesothelioma in the United States alone is approximately 3,200 cases/year [8-11] and expected to rise worldwide [12]. The standard of care for all subtypes is chemotherapy such as cisplatin, pemetrexed, carboplatin, gemcitabine, or doxorubicin [13-15]. In selected specialized centers, a multimodal approach is employed which includes radical cytoreductive surgery followed by radiation, chemotherapy, or targeted therapy. The sequences of treatments is guided by clinical tumor stage or patients' responses and depend on institutional experience [16-19]. Pleural mesothelioma has poor prognosis and patients have a median survival of 4-12 months post diagnosis when treated with chemotherapy [16]. In some cases, although debated, patients treated with multimodal neoadjuvant therapy followed by radical surgery and adjuvant/targeted therapy survive longer for approximately 24 months [20, 21]. Recently, a meta-analysis showed that adjuvant radiotherapy does not improve survival [22].

## Pathogenesis and pathology of malignant mesotheliomas

Mesothelioma is often caused by asbestos exposure [23, 24]. However, in addition to the six fibers collectively called asbestos (the only mineral fibers used commercially in the 1970s), many other mineral fibers (e.g. erionite) that are naturally present in the environment can cause mesotheliomas [23, 25]. Persistent asbestos fiber pressure exerts a slow inflammatory, toxicity, and mutagenic response that can drive mesothelioma [26]. This occurs through altering characteristics attributed to promotion of cell proliferation, high mobility group box 1 (HMGB1) protein secretion [27, 28], sustained angiogenesis [29, 30], and alterations in the expression of redox dependent enzymes (e.g. MnSODs, SODs, catalases and oxygenase) [31-33]. Apart from domestic, environmental, and occupational exposure to asbestos or other carcinogenic mineral fibers, mesotheliomas can also be caused by inherited *BAP1* germline mutations [34]. Moreover carriers of germline *BAP1* mutations are at increased risk of mesothelioma when exposed to asbestos, including low doses that usually are not sufficient to cause cancer [35, 36]. In addition, immune deficiency [37], chronic inflammation [38, 39], ionizing radiation [40, 41], and Simian virus 40 infection have been linked to development of mesothelioma [42-46]. Secondary mesothelioma malignancies may also develop after radiation therapy treatment of lymphomas [47, 48], breast [49], and testicular cancers [50].

To reliably diagnose and determine pathological subtypes of the disease, diagnostic surgery and biopsies of malignant tissues are needed so they can be subjected to further histological examination [51, 52]. Mesotheliomas are classically divided into three pathologic subtypes (i.e. epithelioid, sarcomatoid and biphasic) that are identified via histological and immunohistochemical examinations. These subtypes present distinct morphology and molecular properties (Figure 1B). Epithelioid tumors consist of rounded to cuboidal-shaped cells, account for 80 to 90% of cases [53, 54] and are associated with longer survival. Sarcomatoid forms comprise of 10-20% of diagnosed cases, have spindle shaped cells and give rise to bulky and aggressive tumors [2, 54]. Biphasic tumor subtypes are as rare as the sarcomatoid and contain a mix of epithelioid and sarcomatous tissue [2, 54, 55].

## Genomic alterations of mesotheliomas amenable to targeted therapy

**Figure 2:**
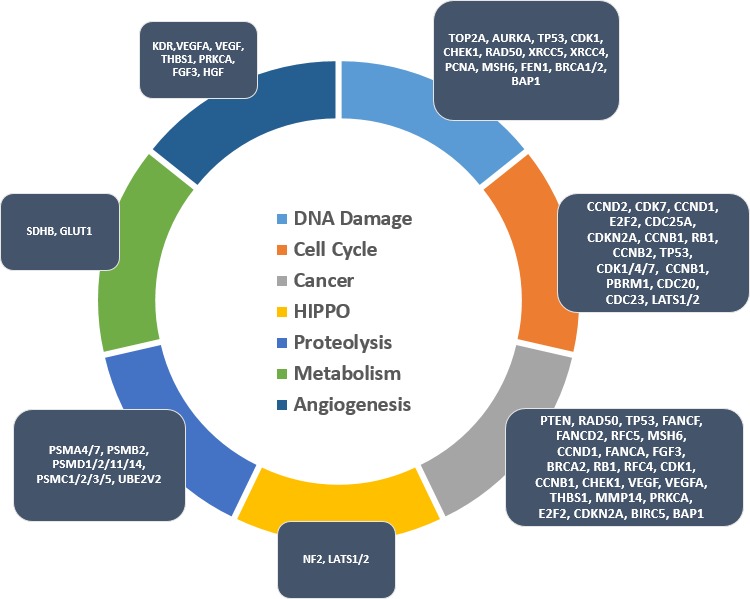
Compilation of common pleural mesothelioma genes and biological pathways: genetic aberrations in pleural mesothelioma and affected downstream signaling pathways, data source extraction from [58, 59, 68–70] Laparoscopy or pleuroscopy show that the healthy intraperitoneal or intrapleural cavities exhibit negative pressures lower than atmospheric pressure [91, 96]. Physiological negative pressure in one study is associated with less than 4% oxygen [97]. Furthermore, mesotheliomas are particularly hypoxic solid tumor masses [85, 92] as evidenced by binding of 2-nitroimidazole or pimonidazole as exogenous hypoxia markers [98, 99] and elevated levels of HIF-1α [100, 101] as endogenous hypoxia marker. Imaging evidence from [F-18] fluoromisonidazole (FMISO) PET-CT scanning confirms hypoxia being integral to mesotheliomas [97].

Although the number of genomic aberrations in mesothelioma is typically lower when compared to other cancers [56], genome-wide profiling reveals enormous complexity in the underlying biology of these tumors [22, 57-65]. The lack of effective therapies and development of resistance is exacerbated by inter and intra-tumor genomic heterogeneities. Genomic aberrations include aneuploidies, point mutations, as well as numerous chromosomal rearrangements that result in deletions, amplification, inversions and translocations [65-67]. Pleural mesotheliomas show considerable genetic variability between morphologic subtypes or patients [58, 59, 68-70], suggesting that a single targeted therapy is unlikely to be beneficial for all patients. Figure 2 lists commonly affected genes in pleural mesothelioma grouped by canonical pathways [58, 59, 68-70]. Such classification is useful to help identify altered cellular mechanisms amenable to therapeutic intervention. Some of the commonly identified mutated or deleted genes in pleural mesotheliomas such as *BAP1 [67], NF2* [71]*, LATS1,2* [63, 72-74], *PBRM1* [67], *TP53* [75-77], *AURKA* [78], *CDKN2A* [79], *RB1* [80], *BRCA2* [81], *CCND1* [82], *SETD2* [83], *SMARCC1 [67]* or *PCNA* [84] are also found in other cancer types. Knowledge of these commonly aberrant genes from other cancer types should be employed in mesothelioma research to help advance precision targeted therapy.

In addition to the cancer cell intrinsic mediators of tumor progression listed above, the tumor microenvironment is known to regulate a variety of genes associated with tumor progression, treatment resistance, and an aggressively metastatic tumor phenotype. The potential role of the tumor microenvironment and tumor hypoxia in driving mesothelioma progression has been under-studied despite evidence that mesotheliomas contain hypoxic tumor cells [85-87]. In this review, we discuss the potential influence of hypoxia on mesothelioma biology and argue that consideration of hypoxia in addition to secondarily affected genes and pathways may permit the design of more specific multi-modal drugs that are activated in hypoxic environments for selective killing of malignant cells [88], improving clinical outcome, and reducing morbidity due to mesothelioma.

## The hypoxic microenvironment of mesotheliomas: clinical and biological evidence

**Figure 3:**
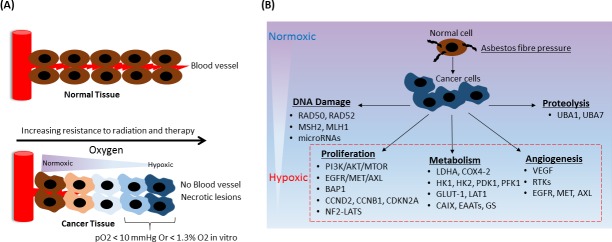
Tumor hypoxia, therapy resistance, and alterations in downstream pathways (A) Tumor hypoxia model of pleural mesothelioma: hypoxia arising in solid tumor tissue regions most distant from the vasculature. (B) Hypoxic cancer cells switch on target genes involved in cell proliferation, DNA damage, metabolism, proteolysis, and angiogenesis pathways leading to cancer cell survival and metastasis. Indicated genes in each pathway are specific to mesotheliomas but also found in other cancers.

Normal tissues exist over a range of oxygen tensions, and low levels of oxygen (hypoxia) are required for a variety of normal processes including embryogenesis, wound healing, and stem cell renewal in the bone marrow. In solid tumors, hypoxia (defined as pO_2_ < 10 mmHg, equivalent to < 1.3% O_2_ *in vitro*) is created when oxygen demand by the proliferating tumor cells exceeds the supply of oxygen provided to the tumor through the bloodstream [63, 89] (see Figure 3A). Tumor hypoxia is a significant barrier to effective treatment since hypoxic tumor cells are known to be resistant to radiation and most chemotherapy, while also promoting the enrichment of tumor cells with stem-like properties [90, 91]. Hypoxia is also associated with tumor progression and metastasis through the activity of the heterodimeric transcription factors hypoxia-inducible factor-1 (HIF-1) and HIF-2's [92], α and β subunits. In normoxic conditions, hydroxylated HIF-1α is ubiquitinated by von-Hippel-Lindau E3 ubiquitin ligase and degraded by the proteasome. However, HIF prolyl-hydroxylases (using oxygen as a co-substrate), inhibited under hypoxic conditions, cannot hydroxylate HIF-1α at its proline residues and thus stabilize HIF-1α [93]. Consequently, HIF signaling cascade activation, due to changes in cellular oxygen concentration, mediates the expression of genes having HIF-responsive elements in their promoters [94]. These genes are implicated in switching and regulating massive pathways such as angiogenesis, metabolism, and survival [95]. Therefore, it is imperative to consider the co-selection of interconnected pathways and their associations with the development of aggressive malignancy.

Cancer cells derive their energy from aerobic glycolytic metabolism for cellular processes based on Warburg's classical observations [102]. Hypoxia, however, triggers a metabolic reprograming of cancers [91] to increase glucose uptake and the flux from pyruvate to lactate. This phenomenon is clinically assessed in mesotheliomas through PET/CT imaging with 2-[^19^F]-fluoro-2-deoxy-D-glucose (F-FDG) tracers [89]. *In vivo,* F-FDG uptake in pleural mesotheliomas shows high correlations with *GLUT1*, *HIF1*, *VEGF*, *CD34*, *Ki67*, and *MTOR* upregulation [93], and poor patient prognoses. Interestingly, *HIF-1* activation increases glucose transport (via GLUT-1) as well as glutamine and L-type amino acid transport (via LAT1) in pleural mesotheliomas [91, 95]. Hypoxia facilitates this switch from oxidative phosphorylation to anaerobic glycolysis [103].

In Figure 3B, we summarize hypoxia-related changes in pathways inherent to solid tumors and commonly aberrant genes found in mesotheliomas. Understanding how mesotheliomas respond to hypoxia and whether selective hypoxia-responsive prodrugs delivered to such tissues are more therapeutically effective remain largely under-investigated and unsolved questions in the mesothelioma field.

## Hypoxia-targeting drugs and strategies for malignant mesotheliomas

Hypoxia as a unique feature of solid tumor biology provokes a need for clinically applicable gene expression signatures and poses a great opportunity for selective antitumor therapies [23, 34]. Apart from the promising direct targeting of HIF-1α in tumors, other potential avenues for therapeutic exploration are prodrugs and enzymes for treatment of cells or tumors under hypoxia. These are listed in table 1, adapted from [1, 104-106].

**Table 1:** Bioreductive prodrugs or polymeric nanoparticles targeting tumor hypoxia

Hypoxia-activated cytotoxins	Examples
Nitro-cyclic compounds	PR-104, TH-302
Aromatic N-oxidases	TPZ, SR4233
Aliphatic N-oxidases	AQ4N
Quinones	Porfiromycin, RH1, EO9
Metal complexes	Cobalt/nitrogen/copper complexes
Polymeric nanoparticles	HR-NPs
**Hypoxia Inhibition**	**Examples**
HIF1 inhibitors	Topotecan, YC-1, PX-478

Therapeutically, hypoxia is exploited in cancer treatment by bioreductive prodrugs such as AQ4N, PR-104, TH-302 or hypoxia-responsive polymeric nanoparticles (containing chemotherapy) [104-107]. These can be likened to ‘Trojan horses’, i.e. normally inactive drug derivatives that undergo bioactivation via enzymatic or chemical transformations [85, 88, 107] under hypoxic conditions. Other strategies explored to date are hypoxia-responsive polymeric nanoparticles, magnetic nanoparticles, small molecule inhibitors, or hypoxia-triggered prodrug micelles that carry and selectively release therapeutic agents in the hypoxic microenvironment of the tumors. Several surrogate markers for recognizing tumor hypoxia are hypoxia-related endogenous proteins (GLUT-1 and CA-IX due to presence of HIF-responsive elements in promoters) as well as exogenous bioreductive hypoxia drugs (Tirapazamine, PR-104, TH-302) [108-110].

Along with well-known GLUT-1 regulation, carbonic anhydrases (CA), large family of zinc metalloproteases, are strongly upregulated by HIF-1 and are required for maintenance of pH, proliferation, and metastasis [111, 112]. CA-IX is a membrane associated isoform showing extensive diversity in tissue distributions and in subcellular localizations [71]. It is an established endogenous marker of hypoxia, and particularly overexpressed in VHL mutated clear cell renal cell carcinomas [113, 114], mesotheliomas [115, 116], kidney [15], as well as other hypoxic solid tumors [113]. CA-IX expression is correlated with poor patient prognosis, metastasis, and therapy resistance [5, 71, 117]. This is particularly interesting as more than 90% of mesotheliomas are positive for CA-IX [95, 115, 118] and so one would expect its inhibition would limit pH-driven growth and metastasis. Monoclonal antibodies and small molecule inhibitors specific to CA-IX are being investigated for potential targeted therapeutics in the pre-clinical studies. Regardless, the sequence of administration in combination with other chemotherapies in mesotheliomas warrants careful investigation. Further, the modest improvements in disease-free survival following hypoxia-targeted therapies in over 30 clinical trials [119, 120] demonstrates the need for considering tumor heterogeneity, hypoxia assessment, and patient stratification prior to therapy.

## Hypoxia-induced angiogenesis and mesothelioma pathogenesis

The vasculature delivers oxygen and nutrients to all cells within the body. Hypoxic regions trigger HIF-related pathways that are key regulators of sprouting angiogenesis via modulating vascular endothelial growth factor (VEGF) [121]. VEGF-induced pro-survival pathways in solid tumors is a pivotal and early event in the development of metastatic malignancies [122]. Strikingly, VEGF along with multiple RTKs essential for VEGF-mediated angiogenesis, including epidermal growth factor receptor (EGFR), MET, and AXL, are activated in pleural mesothelioma cell lines and tumors [123-126]. Although clinical trial results for EGFR inhibitors in mesothelioma have not been released, concurrent inhibition of various activated RTKs with pro-apoptotic and anti-proliferative effects in mesothelioma cell lines have paved the way to such trials [127]. It is noteworthy that hypoxia induces the activity of tyrosine kinase inhibitors [128]. This selective activation under hypoxia would beis therefore an interesting avenue to explore [129] compared to conventional cytotoxic drugs that affect all cells. There are numerous studies using antiangiogenic agents Bevacizumab and Sunitinib (VEGF inhibitors), and Sorafenib (tyrosine kinase inhibitor) for cancer therapy with beneficial results for patients with other tumor types [101, 130]. A randomized phase II trial has not significantly improved progression-free survival in pleural mesothelioma patients [131], arguably for the same reason that patients aren't stratified based on tumor hypoxia assessments [132]. Anti-VEGF inhibitors do elicit response in primary tumors but evasive resistance develops and results in aggressive regression in glioblastomas [99, 133]. Antiangiogenic effects of agents such as Bevacizumab in combination with hypoxia-activated prodrugs or HIF-1 inhibitors and standard chemotherapies, however, have served as attractive strategies to target the hypoxic tumor microenvironment in triple negative breast cancer and gliomas [115, 134, 135]. VEGF inhibitor (NCT00651456) in addition to chemotherapy in pleural mesothelioma patients is currently in phase III clinical trials with longer survival success [J Clin Oncol 33, 2015 (abstract 7500)]. A consideration of sequential multi-modal regimens of therapy is arguably the reason behind lack of improvements in patient survival rate. A sequential multi-modal regimen of chemotherapy, VEGF and HIF inhibitors in addition to other secondary hypoxia-activated pathways would require thorough preclinical and clinical investigations.

## Hypoxia-induced proliferation inMesotheliomas

PI3K/AKT/MTOR is an oxygen and energy-sensing pathway essential for regulation of cell cycle progression and cell proliferation [136], and closely associated with hypoxic signaling (e.g. mediating HIF-1 regulation) [137, 138]. Epithelioid-type pleural mesothelioma cells show activation of PI3K/AKT/MTOR signaling [139-141]. PI3K/AKT/MTOR signaling is partly dependent upon coordinated activation of multiple receptor tyrosine kinases (RTKs), such as *EGFR*, *MET* or *AXL* [127, 140]. Interestingly, signaling through these RTKs has also been found to be altered in 8 out of 9 pleural mesothelioma cell lines and 6 of 12 mesothelioma biopsies [136]. There are currently phase I, II, and III clinical trials evaluating AXL inhibitor (BGB324) in colon cancer [142], MET inhibitor (INC280) in papillary renal cell cancer, and EGFR inhibitor (NCT02206763) in non-small cell lung cancer and so re-purposing the successful ones for mesothelioma therapy will be essential. Further, although a direct link between PI3K/AKT/MTOR and hypoxia has not been established in mesothelioma, an integrated multi-modal approach to target pathways affecting cell proliferation and survival under hypoxia remain to be investigated (Table 2).

**Table 2:** Clinical Trials in Pleural Mesothelioma

Clinical Trial ID	Phase	Agent Tested	Mechanisms of Action
NCT01675765	I	CRS-207	Immunotherapy Against Tumor Associated Antigen Mesothelin
NCT01870609	II	VS-6063	Tumor NF2 Antagonist
NCT02071862	I	CB-839	Glutaminase Inhibitor
NCT00685204	II	TL139	Taxane
NCT02372227	I	VS-6063	Dual PI3K/mTOR Inhibitor
NCT01655225	I	LY3023414	Inhibit CYP3A4-mediated Metabolism
NCT01997190	I	AdV-tk	Adenovirus-mediated Herpes Simplex Virus Against Thymidine Kinase
NCT00996567	II	Cetuximab	Antibody Against EGFR
NCT01938443	I	GSK2256098	FAK Inhibitor
NCT01358084	II	NGR-hTNF	Vascular Targeting Agent
NCT01211275	II	Axitinib	VEGF Angiogenesis Inhibitor

A tumor suppressor gene most commonly deleted or mutated in mesotheliomas (∼60% of cases) is *BRCA1* associated protein-1 (BAP1), a C-terminal family of deubiquitinating enzymes (DUBs) linked to DNA damage repair regulation [36, 63, 67, 83, 143-145]. *BAP1*'s function has been implicated in various other cancer types such as uveal melanoma [113], clear cell renal cell carcinoma [114], and cutaneous melanocytic tumors [113, 116]. Although *BAP1*'s crystal structure has been solved [92], a therapeutic drug for patients carrying mutations of *BAP1* has not been developed. Moreover, cell cycle related genes often found mutated in pleural mesothelioma and regulated by hypoxic stress [132, 135] are cyclin-dependent kinases (such as ∼15-45% incidence in deletions of *CDKN1,2A*) [22, 83, 146, 147]. Using drugs that target these genes in combination with hypoxia-specific cytotoxins warrants pre- and clinical investigations. Of note are CDK4 inhibitor (palbociclib) trials for non-mesothelioma cancer patients under way that could be repurposed for mesotheliomas. Interestingly, BAP1 inactivation is associated with carbonic anhydrase 9 (CA-IX) expression [15].

Additionally, two important genes consistently found mutated or inactivated in pleural mesotheliomas are neurofibromin or merlin (*NF2,* with ∼ 45% incidence of aberration), a negative regulator of E3 ubiquitin ligase, and the Large Tumor Suppressor kinase 1/2 (*LATS1/2,* with ∼ 30% incidence of aberration), two components of the Hippo pathway [22, 83, 148]. Both *NF2* and *LATS2* can be regulated by hypoxia [149] but this particular link has not been studied in mesothelioma. The co-targeting of both *LATS2* and *NF2* delivered into the hypoxic tumors may prove more potent and call for thorough clinical investigations (table 2).

## Hypoxia-induced DNA damage repair in mesotheliomas

Typically, as an adaptive response to hypoxia, tumors increase genetic instability by down regulating DNA repair genes such as *MLH1, MSH2, RAD50-2* and activating ATM and ATR DNA damage checkpoint pathways [121, 150, 151]. The homologous and non-homologous recombination as well as mismatch are inhibited under hypoxia, increasing unrepaired replication errors and double stranded breaks [117]. More specifically, cells under hypoxia and/or reoxygenation are most sensitive to loss or inhibition of *CHEK1*, *ATM*, and *ATR* [152]. In pleural mesotheliomas, tumor suppressor genes *MSH6* (heterodimer partner of *MSH2*) and *RAD50* are highly overexpressed, especially post-chemotherapy [30]. Further, *CHEK1*, required for checkpoint mediated cell cycle arrest in response to DNA damage, is overexpressed [30] in pleural mesotheliomas. A *CHEK1* inhibitor, LY2606369, is currently used in clinical trials for breast cancer patients with *BRCA1/2* mutations and could be re-purposed for treating pleural mesothelioma malignancy. Other DNA damage repair genes deregulated in mesotheliomas include Fanconi anemia group D2 *(FANCD2)*, *RAD21* and *RAN [153]*. Thus, co-targeting DNA damage genes in addition to chemotherapy may improve patient survival.

*RAD52*, another key gene involved in homologous recombination repair, is important for chemotherapy resistance and can be translationally repressed by miR-210 [150], a microRNA regulated by hypoxia. Additionally, there are many microRNAs identified to date to be associated with poor survival in mesotheliomas (miR-210, mir-126, miR-125a-5p, miR-484, miR-320, and let-7a, miR-29c, miR-16, miR-31, miR-34 [154-156], miR-141, miR-200a, miR-200b, miR-200c, miR-203, miR-205, and miR-429 [157], and miR-193, miR-200, and miR-192 [158, 159] with diagnostic confidence). Remarkably, hypoxic cells also show lower expression levels of miR-141 [86]. The therapeutic potential of using microRNA mimics [160, 161] in conjunction with hypoxia-responsive polymeric nanoparticles *in vivo* demands closer investigations and gives countless potentially druggable targets for the development of innovative cures.

## Hypoxia-induced proteolysis in mesotheliomas

Under cellular stress conditions (e.g. low nutrient or oxygen levels), alteration/induction of proteasome and autophagic lysosome degradation pathways occurs as an adaptive response to mitigate the new cellular energy demands [162, 163]. Specifically, severe hypoxia increases unfolded protein response (UPR), causes accumulation of unfolded proteins in the endoplasmic reticulum (ER), and leads to more stress [88, 164]. Further, UPR can subsequently activate autophagy to alleviate stress via inducing apoptosis, increasing cell survival and proliferation [165]. Interestingly, the ubiquitin-proteasome pathway is differentially regulated in epithelioid versus biphasic pleural mesotheliomas [166]. Epithelioid pleural tumors have lower levels of ubiquitin specific proteases and higher levels of ubiquitin-activating enzyme E1, which associates with long term survival [167]. Short-term survivors have higher proteasome subunits [168]. Higher levels of Cullin 4A, an ubiquitin ligase E3, are reported in pleural mesotheliomas [169]. The selective ER stress-inducing agents and UPR inhibitors can be particularly promising in mesothelioma targeted therapy. For instance, inhibiting the UPR (with MG132 and PSI) results in apoptosis and inhibition of invasion in malignant pleural mesothelioma cells [170, 171]. Proteasome inhibitor, Bortezomib is currently in phase II clinical trials in combination with chemotherapy showing improved patient outcome for pleural mesothelioma [172]. Intriguingly, the link between hypoxia and proteolysis has not been thoroughly investigated in mesotheliomas.

## CONCLUDING REMARKS

Some of the outstanding questions in the field of malignant mesothelioma biology include deciphering which molecular events are involved in the genesis of mesotheliomas and would this inter and intra-heterogeneity among tumors change from onset to progression? Understanding the genomic landscape of mesotheliomas and integrating that knowledge for designing optimal and tailored therapeutic strategies is critical for improved patient outcome. Another important aspect is to understand whether and how the microenvironment of malignant mesotheliomas contributes to changes in oxygen permeability, nutrition and pH of tumors [173] and whether sustained *HIF* induction is necessary for continued growth and survival of these tumors.

Hypoxic cells within the tumor mass are distant from blood vessels, resistant to most anticancer drugs, and present a major obstacle to delivery of targeted therapies. In this review, we suggest that the hypoxic environment of solid tumor mesotheliomas can be used as the Achilles' heel for targeted drug delivery. We ask whether new therapies for pleural mesothelioma, such as those being used in clinical trials worldwide (Table 2), prove more effective if tumor-hypoxia is carefully assessed and therapies are administered in combination with hypoxia-based therapies.

How we address the challenge of individualized and direct assessment of oxygen pressure within mesotheliomas, correlate that measurement to tumor function, and incorporate it as part of a standard of care remain outstanding areas for investigations.
